# Movement assay for the undergraduate neuroscience laboratory

**DOI:** 10.1016/j.ohx.2020.e00094

**Published:** 2020-01-27

**Authors:** Cody R. Townsley, Joseph M. Breza, Thomas G. Mast

**Affiliations:** a Program in Neuroscience, Eastern Michigan University, Ypsilanti, MI, USA; b Department of Biology, Eastern Michigan University, Ypsilanti, MI, USA; c Department of Psychology, Eastern Michigan University, Ypsilanti, MI, USA

**Keywords:** Piezoelectric, Movement, Behavior, Flight

## Abstract

Described is a design for easy-to-construct apparatus that measures movement of flying insects suitable for the undergraduate teaching laboratory. The system does not require the purchase of specialized scientific equipment or software. The apparatus can be constructed and operated without advanced knowledge in electronics or programming. The goal of this apparatus was to expand upon previous research detecting insect flight in response to radiation. We improved upon the quantification and resolution of flight across differing intensities of white light. All of this was achieved using low-cost and commonly available materials and open-source software to collect and analyze data. The only substantial prerequisites for this design are a PC with a 3.5 mm microphone input and an understanding of basic electrical connections. The apparatus was validated with comparative physiological data from two different species of butterfly.

## Specifications Table

**Table:** 

Hardware name	Butterfly Movement Assay
Subject area	Neuroscience
Hardware type	Measuring physical properties and in-lab sensors
Open Source License	GNU General Public License
Cost of Hardware	*$548.73*
Source File Repository	https://doi.org/10.17605/OSF.IO/XM96N

## 1. Hardware in context

Behavioral neuroscience assays appropriate for undergraduate laboratory courses are not in use at many universities [1] due in large part to the cost of materials needed to conduct feasible experiments [2]. Yet, universities are creating course that integrate physics, electrical engineering, computational science, and hardware-software design as a way to prepare future neuroscientists [3]. Thus, to ensure that future neuroscientists are prepared, it is important that these areas are adequately taught while remaining inexpensive and user efficient. Therefore, we set out to create equipment that can be used with both commercially-available and open-source resources.

Many researchers are currently developing inexpensive and user efficient tools that introduce fundamental concepts of neuroscience to students. For example, the introduction of the Backyard Brains SpikerBox has streamlined the process of recording action potentials with a few simple components [4] and has already been used in laboratory-class settings [1]. Further, other scientists are currently developing open-source behavioral neuroscience equipment including operant conditioning boxes [5,6]. Previously, we have published an experimental assay used to count the frequency, duration, and pattern of mice licking a spout [7].

In the present study, we made revisions of an experiment conducted by Smith et al. (1963) [8] that measured moth flight responses to x-ray radiation. Smith et al. (1963) measured flight occurrence to find sensory-threshold in dark-adapted moths. We expanded upon this experiment by creating a hardware-software configuration that introduces visual stimuli to dark-adapted insects and allows for the quantification their movement in an intensity-response fashion. Using a flying invertebrate as a teaching organism reduces the number of vertebrate animals used in research [9]. The present hardware design aimed to: 1) digitize movement through voltage differentials from a piezoelectric; 2) ensure motor activity was due to stimulus presentation; 3) distinguish movement initiation and motor output quantification to different stimuli; 4) be user-friendly for novices in an educational setting. The experimental assay utilizes inexpensive and readily obtainable equipment; piezoelectrics, Backyard Brains amplifier, a computer with a soundcard, open-source recording (Audacity) and analysis software (R), and commonly used educational equipment (iWorx).

## 2. Hardware description

- 3.5 mm RCA to 3.5 mm RCA
- Backyard Brains Amplifier (BYB). BYB-Part#: SB001. Rev: 1.3b.
- 6.35 mm-RCA-to-two-pin (Provided with BYB amplifier)
- IWorx TA
- 4 Solderless Breadboards
- Arduino Uno Microcontroller (Arduino online store; Code: A000066)
- USB Data Sync Cable (Provided with the Arduino Uno components)
- Piezoelectric Sensor (TE Connectivity DT series Part#: CAT-PFS00004)
- Wooden Dowel
- Hot Glue
- Straw (that fits over the wooden dowel snuggly) or a disposable transfer pipette
- 2 Styrofoam Cooler
- Insulation (Cut to fit into the opening of the Styrofoam cooler)
- 5 Super Bright White 5 mm LED’s (Adafruit.com; Product ID: 754)
- 6 resistors (1MΩ, 100kΩ, 10kΩ, 1kΩ, 100 Ω, Arbitrary)
- 1 100 mL beaker
- ~43 jumper wires (various; male-to-male male-to-female female-to-female)
- 1 Tactile Button Switch (Adafruit.com; Product ID: 367)
- 9 V battery
- Dental Wax
- Hotplate

## 3. Design files

**Table:** 

File name	Type	License	Location
ButtonSwitch	.pdf	GNU General Public License	https://doi.org/10.17605/OSF.IO/XM96N
HabituationChamber	.pdf	GNU General Public License	https://doi.org/10.17605/OSF.IO/XM96N
LEDBreadBoardSchematic	.jpg	GNU General Public License	https://doi.org/10.17605/OSF.IO/XM96N
PiezoSchematic	.jpg	GNU General Public License	https://doi.org/10.17605/OSF.IO/XM96N

## 4. Bill of materials

**Table:** 

Designator	Component	Amount	Component Cost ($)	Total Cost ($)	Source
See Build Instructions	Neuron SpikerBox Bundle	1	$129.99	$129.99	https://Backyardbrains.com/products/spikerboxBundle
	Half-size Breadboard	4	$5.00	$20.00	https://www.adafruit.com/product/64
	Arduino Uno Rev3	1	$22.00	$22.00	https://store.arduino.cc/usa/arduino-uno-rev3
	Piezo Vibration Sensor - Large	1	$2.95	$2.95	https://www.sparkfun.com/products/9196
	Wood Applicator Stick	1	$10.50/1000	$10.50	https://www.fishersci.com/shop/products/hospital-wood-applicator-stick-economy-grade/22029669#?keyword=applicator+sticks
	Super Bright White 5 mm LED	5	$6.95/25	$6.95	https://www.adafruit.com/product/754
	Styrofoam Cooler	1	$29.95	$29.95	https://www.amazon.com/Polar-Tech-205C-Insulated-Shipper/dp/B007PB0ZK2/ref=sr_1_1?keywords=styrofoam+cooler+polar+tech+205c&qid=1579900469&s=industrial&sr=1-1
	E12-Series Resistor Pack	1	$8.12	$8.12	https://www.jameco.com?z?K-RES-E12-Velleman-E12-Series-1-4-Watt-5-Resistor-Pack-610-pcs_2131039.html
	Sound Proof Foam	1	$16.95	$16.95	https://www.amazon.com/Soundproofing-Acoustic-Studio-Foam-Panels/dp/B06XRJYSV2/ref=sr_1_6?keywords=12+acoustic+panels+studio+wedges&qid=1579900844&s=industrial&sr=8-6
	Jumper Wire	1	$1.95	$1.95	https://www.digikey.com/product-detail/en/adafruit-industries-llc/1953/1528-2233-ND/7241478
	Tactile Button Switch	1	$2.50	$2.50	https://www.adafruit.com/product/367
	Dental Wax	1	$9.99	$9.99	https://www.amazon.com/Zorvo-Dental-Plate-Utility-Supply/dp/B01N4J79U/ref=sr_1_9?keywords=dental+wax+sheets&qid=1557953038&s=gatewt&sr=8-9
	Hot Plate	1	$269.92	$269.92	https://www.amazon.com/Corning-6795-400D-PC-400D-Digital-Pyroceram/dp/B004DGID7Y/ref=sr_1_2?keywords=corning+hotplate&qid=1579901270&sr=8-2

## 5. Build instructions

### 5.1. Electrical connections

Three sets of electrical connections need to be made:

1. Computer soundcard to piezoelectric or IWorx TA to piezoelectric
2. Microcontroller to LED lightboard
3. Microcontroller to tactile button switch

### 5.2. Piezoelectric sensor

**Fig. 1.**
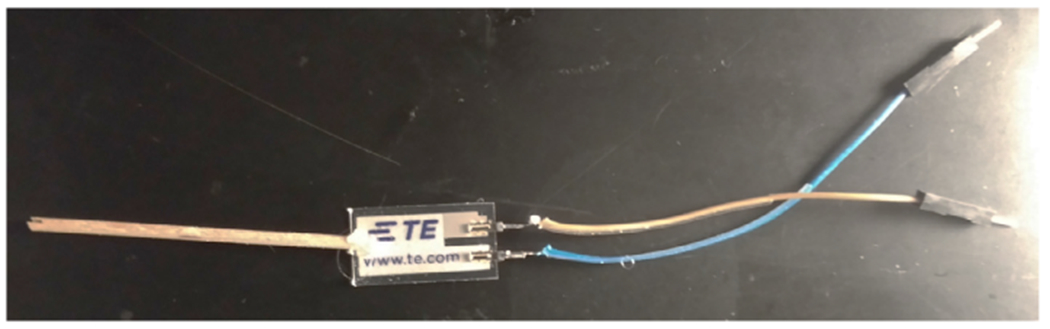
Example of piezoelectric construction. Male-ended jumper wires soldered to each PCB trace of the piezoelectric. Small wooden dowel attached with hot glue to the other end.

A piezoelectric film was chosen to transduce insect movement into an analog (i.e. waveform) signal. Piezoelectric materials generate voltages in response to force and the size of the voltage is proportional to the size of the applied force [11]. Practically, the piezoelectric sensor must hold the insect and be able to attach to the hardware. First, a stripped male-ended jumper wire was soldered to each PCB trace of the piezoelectric. Next, a small wooden dowel was glued to the end as a place of attachment for the insect (Fig. 1).

### 5.3. Computer soundcard to piezoelectric

**Fig. 2.**
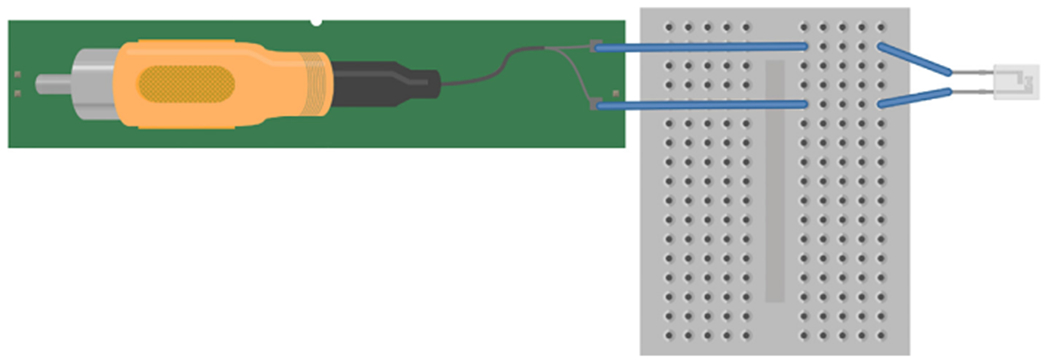
Schematic of piezoelectric wiring. Two pins coming from the 6.35 mm RCA were connected to separate terminal strips in a breadboard. The male-ended jumper from the piezoelectric were connected to corresponding terminal strips. Schematic made with Fritzing (www.fritzing.org).

The amplifier was connected to the computer with 3.5 mm RCA to 3.5 mm RCA cable (Fig. 2). One 3.5 mm RCA was plugged into the input/output headphone jack of the computer and connected to the computer soundcard. The other 3.5 mm RCA end was plugged into the output of the BYB amplifier. The 6.35 mm RCA (provided with BYB amplifier) was connected to the input port of the BYB amplifier. There are two active electrode pins coming from the 6.35 mm RCA, each was plugged into a separate terminal strip of a breadboard. The male-ended jumper wires soldered to each PCB trace of the piezoelectric were placed in corresponding terminal strips to make connection to the pins of the 6.35 mm RCA.

### 5.4. IWorx TA to piezoelectric

The piezoelectric sensor can be connected directly to the iWorx TA module with BNC-to-alligator-clip connectors (i.e. omitting an amplifier). The BNC connector was connected to a channel of the IWorx module, the alligator clips were clipped to each male-ended jumper wire soldered to the PCB trace of the piezoelectric (cable comes with the TA module).

### 5.5. Computer to microcontroller

The computer was connected to the microcontroller with the USB cable. This connection was used to control the LED’s of the lightboard and the tactile button switch (discussed below).

### 5.6. Microcontroller to LED lightboard

**Fig. 3.**
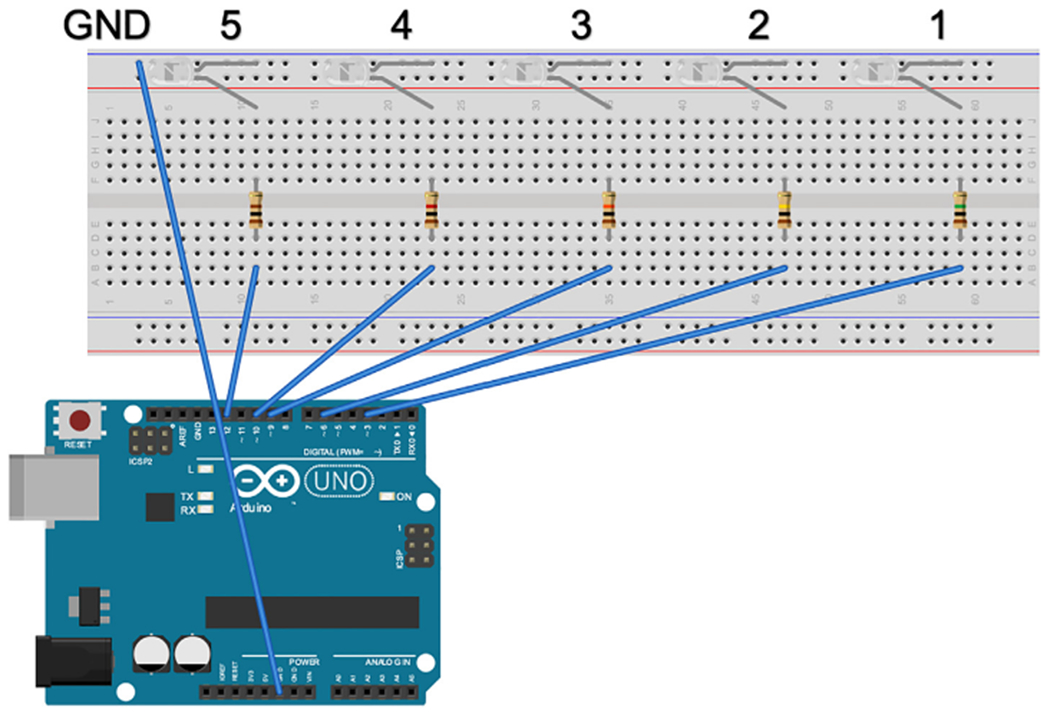
Schematic of the LED lightboard. The power ground pin of the Arduino Uno was connected to the ground bus strip of a breadboard (labeled GND). The cathode (long pin) of each LED was connected to the ground bus strip (GND). The anode (short pin) of each LED was connected to a numbered terminal of the breadboard. A resistor was connected to each LED in accordance to brightness desired for the LED. The resistor was connected to another numbered terminal of the breadboard across the notch. A corresponding coded digital pin of the Arduino Uno was connected to each resistor through a numbered terminal (1–5). Schematic made with Fritzing (www.fritzing.org).

The power ground pin of the Arduino Uno Microcontroller was connected to the ground bus strip of the breadboard (Fig. 3). The cathode of each LED was connected to the ground bus strip and the anode was connected to a terminal strip. A resistor was connected to the terminal strip of each LED, the other end of the resistor was connected to another terminal across the notch. The digital pins corresponding to each LED were connected to the other end of each resistor through the terminal strip.

The brightness of each LED was measured in footcandles (fc) using a light meter (Extech; #401027). The LED’s used in the proof of principal experiment had a brightness of; <0.1fc, .3fc, 3.5fc, 57.1fc, and 297fc, corresponding to 1MΩ, 100KΩ, 10 KΩ, 1 KΩ, and 100 Ω resistors, respectively.

### 5.7. Microcontroller to tactile button switch

**Fig. 4.**
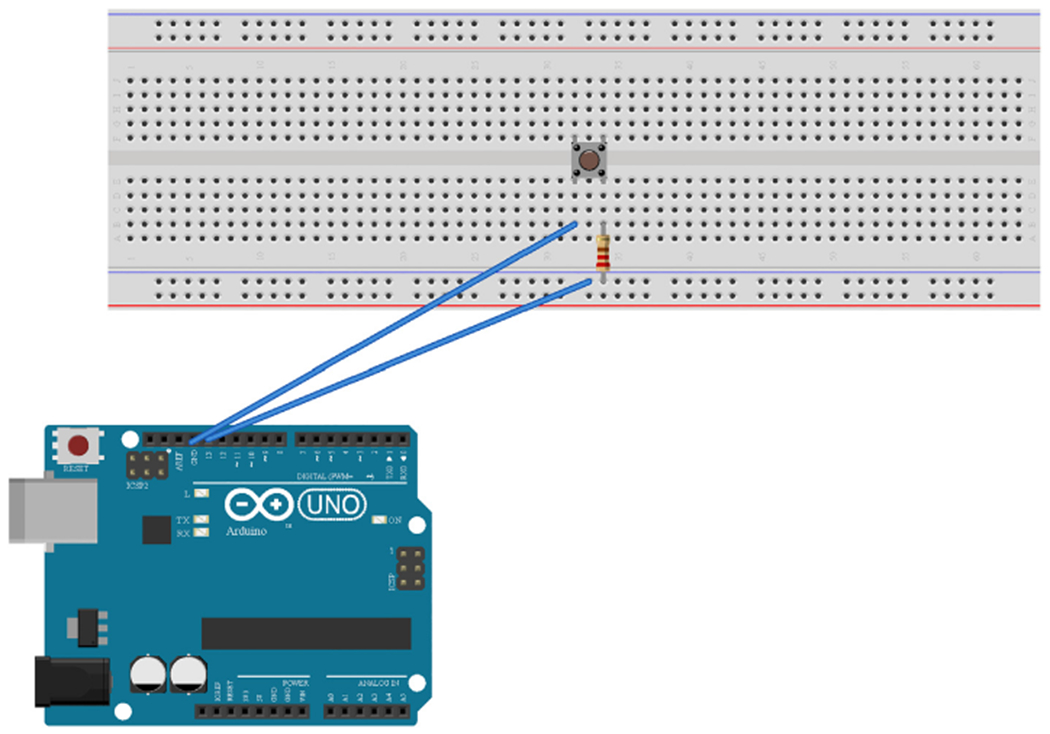
Schematic of the tactile button switch. The legs of the button were placed across a breadboard notch. The digital ground of the Arduino Uno was connected through a terminal of the breadboard to a pin of the button switch. The digital pin coded to control the button was connected to a bus strip. A resistor was connected to the same bus strip and connected to a second pin of the tactile button switch. Note: the resistance of the resistor used for this connection is arbitrary. Schematic made with Fritzing (www.fritzing.org).

A switch circuit was built to control the lightboard. The tactile button was connected across the notch of another breadboard (Fig. 4). The digital ground pin of the Arduino Uno was connected to a terminal connected to one of the tactile button switch pins. A digital pin was connected to a bus terminal and a resistor was connected from the bus terminal to a second pin of the tactile button switch. Each pin of the button acts as an active pin. Connection is completed between the pins once the button is pressed, thus completing the circuit between the two digital pins of the microcontroller.

### 5.8. Habituation chamber

**Fig. 5.**
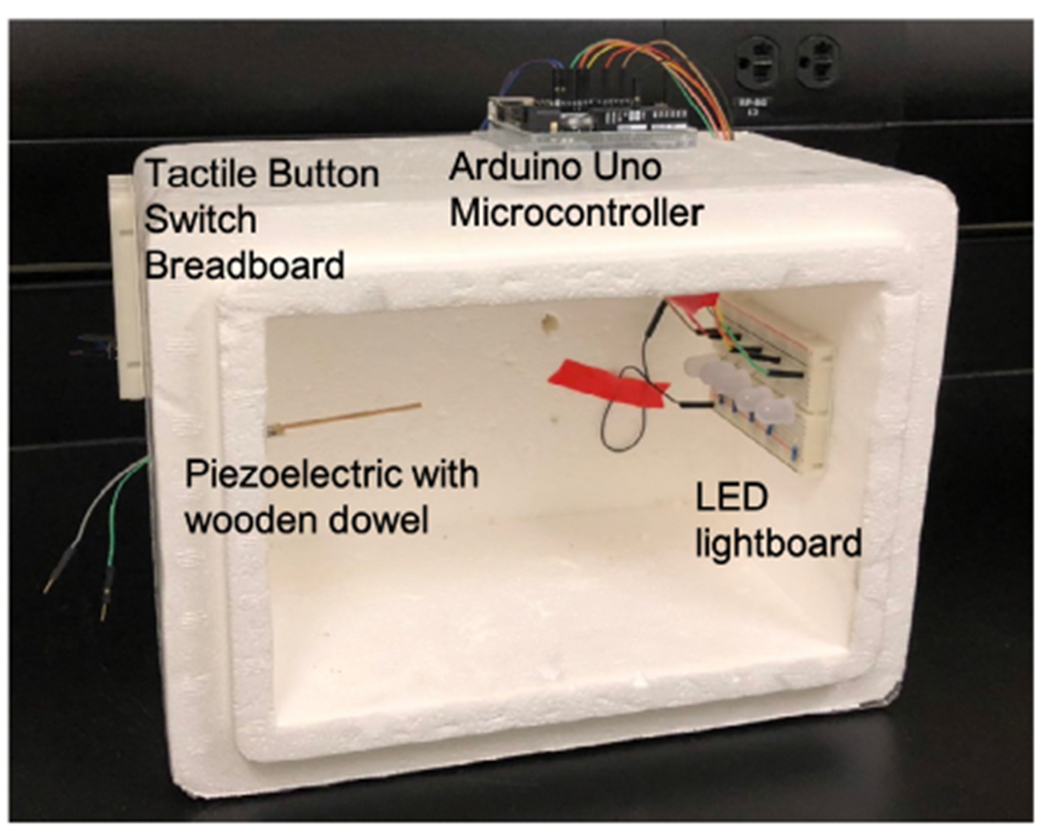
Example of habituation chamber construction. Note the location of the piezoelectric sensor fitting part-way into the chamber wall. Also note the lightboard inside the chamber, while the Arduino and tactile button are outside the chamber.

The experiment was conducted inside a dark chamber so that the insects could be dark adapted. To create a habituation chamber, the sensor, light-board, and button were affixed to a suitable box (Fig. 5). The piezoelectric with wooden dowel was fed through a hole in the side wall of the habituation chamber. Wires from the microcontroller were fed through a hole in the opposite wall to the LED lightboard inside the habituation chamber. A piece of foam insulation was cut to fit into the opening of the habituation chamber for dark adaption (not pictured). A tactile button switch was connected to the microcontroller and mounted on the outside of the habituation chamber

### 5.9. Custom software

Arduino IDE software was used to code digital pins of the microcontroller for the lightboard. The tactile button switch (Fig. 4) was coded to initiate the LED sequence after the button was pressed (Pin 13 and GND of digital pins). The LED’s were coded to turn on and off in sequence according to their brightness in ascending order, with highest resistance/lowest intensity initiating first, and lowest resistance/highest intensity initiating last (Pin 3; 1 MΩ, <.1fc. Pin 6; 100 KΩ, 0.3 fc. Pin 9; 10 KΩ, 3.5 fc. Pin 10; 1 KΩ, 57.1 fc. Pin 12; 100 Ω 297 fc).

**Fig. 6.**
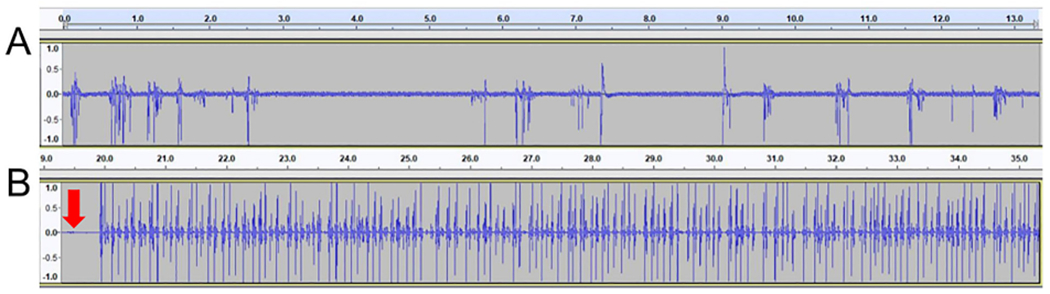
Example piezoelectric recordings in Audacity. (A) Sample recording using a flick test using a 0.04 g (0.39 mN force) Von Frey filament. (B) Sample recording of Painted Lady flight during experiment. The downward red arrow marks LED illumination. (For interpretation of the references to color in this figure legend, the reader is referred to the web version of this article.)

Voltages collected using the BYB amplifier was recorded using the freeware Audacity, and analyzed using the open source program R. Sections of recordings after the illumination of the LED’s were exported as a .wav files. WingBeat.R was used to analyze each .wav file (Fig. 6). Voltage differentials displayed in the tracks of the Audacity file were analyzed by a custom R script WingBeat.R. WingBeat.R generates a count for each time a manually set dB threshold is passed. WingBeat.R was generated by modifications made to the R script used by Raymond et al. [7]. Full documentation is on the OSF website. Alternatively, piezoelectice voltages were recorded using the iWorx TA system and analyzed using Labscribe 3 (see operating instructions).

## 6. Operating instructions

### 6.1. Step-by-step hardware instructions

1. Connect all hardware to computer.
2. Turn on the BYB.
3. Make sure the Arduino Uno “ON” LED is illuminated.
4. Open Arduino IDE program with code provided.
5. Perform a test of the lightboard. (lower each “delay” by a factor of 10 to speed up the process).
6. Open Audacity and start a recording.
7. Perform a flick test of the piezoelectric.
8. Cut ~3 cm of straw section. Cut a coronal section of straw about the size of the body of the insect you wish to connect to the movement assay.
9. Acquire the insect and pin back wings, exposing the midsection.
10. Melt a small amount of dental wax on a hotplate. Using the open section of straw, scoop up the melted wax.
11. Press the straw to the insect’s midsection and hold until dental wax hardens.
12. Slide the straw over the wooden dowel. If the piezoelectric bends under the weight of the insect place a stabilizer under the wooden dowel.
13. Place foam insulation into the opening of the behavior chamber and allow the insect to dark adapt. Using the recording software, you can see when the insect has stopped moving.
14. Once the insect has dark adapted, press the tactile button switch to initiate LED sequence.
15. Note the time that the button was pressed and when each LED is coded to turn on.

The same instructions can be used for both the BYB and iWorx TA.

### 6.2. Signal processing

To test the flexibility of the hardware, the analog voltage was digitized, visualized, and analyzed with two different configurations. An inexpensive method used a BYB SpikerBox Amplifier, computer sound card, Audacity, and analyzed by Wing-Beat. R. A more expensive, but user friendly, set-up used the iWorx TA to digitize the signal and Labscribe3 software to visualize and analyze the software. Access to iWorx was provided through Eastern Michigan University’s Neuroscience program. Audacity and iWorx produced similar recordings (Fig. 8).

**Fig. 7.**
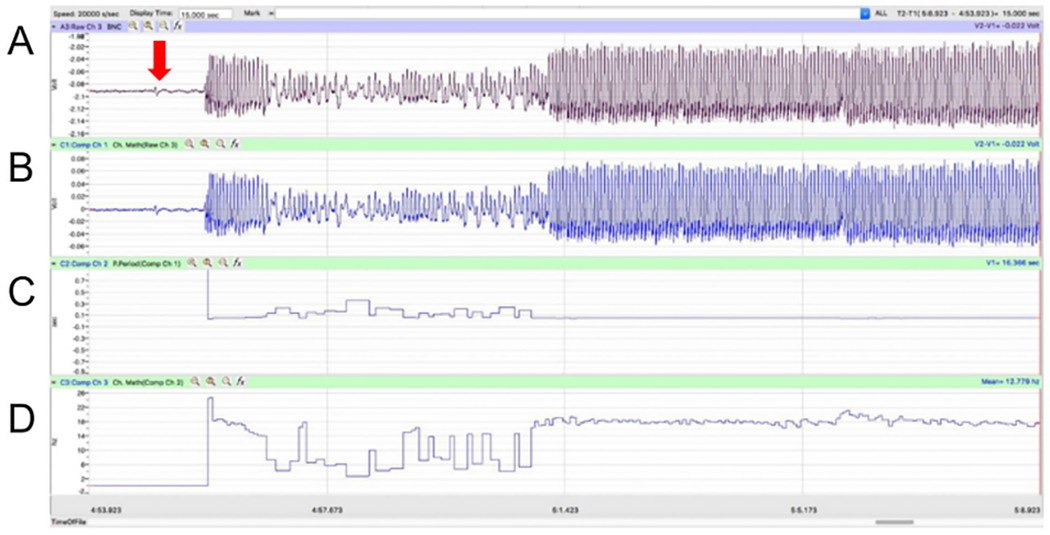
Example piezoelectric recordings in Labscribe3. (A) Deflections in the piezoelectric were recorded and digitized through the iWorx TA module and displayed using Labscribe3 software. (B) Raw recordings were offset to have a zero baseline. (C) The period was calculated from the zero-baseline channel. (D) The reciprocal of the period was plotted to produce a rate of peak deflections per second (i.e. hertz).

**Fig. 8.**
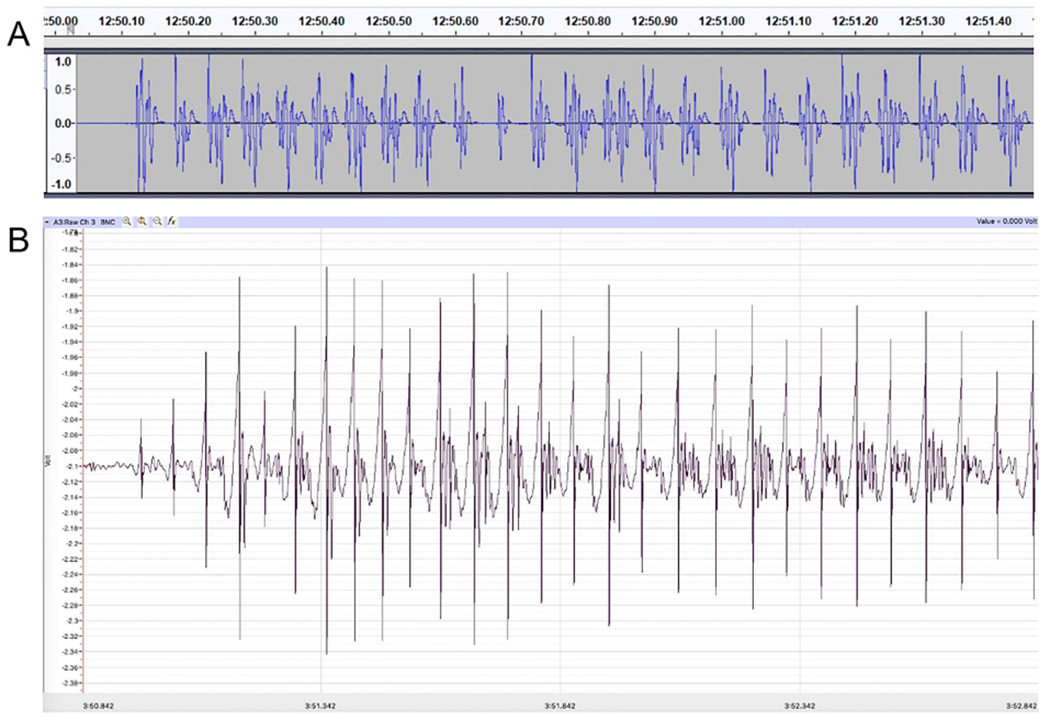
Example deflections from two painted ladies following 57.1 fc illumination. (A) Deflections recorded using the BYB amplifier and Audacity. (B) Deflections recorded using the iWorx TA module and Labscribe3. Each system produced a rhythmic, complex wingbeat.

Voltage collected using the the IWorx TA machine was recorded and analyzed using Labscribe3 software in a classroom setting (Fig. 7). The raw recording was first offset in a new channel to have a zero baseline (function: channel math). Then the period was calculated in an additional channel (function: period). Finally, the reciprocal of period was plotted in the last channel to get rate in hertz (function: channel math).

## 7. Validation and characterization

As a proof of concept, we conducted an experiment to measure light-induced flying of two different species of butterfly: cabbage whites and painted ladies. Cabbage whites and painted ladies were chosen as they are common models of insect visual processing. Cabbage whites’ eyes have peak sensitivities at 360, 450, 540, and 620 nm, and will produce feeding movement based on white light introduction [11,12]. In contrast, painted ladies eyes have only three peak sensitivities at 360, 470 and 530 nm [13] and cannot discriminate between 440 and 620 nm [134]. The white LEDs selected for this experiment have a peak around 620 nm. Therefore, we hypothesized that the cabbage whites would show an increased sensitivity to these LEDs compared to the painted ladies. Statistical analysis of behavior (ANOVA and Chi-squared) and graphs were generated in Prism 8 (GraphPad). Alpha was set to 0.05.

To test this hypothesis, insects were attached to the piezoelectric (N = 5 cabbage whites; N = 13 painted ladies) and allowed to dark adapt for 15 min within the habituation chamber. The code was then initiated and time stamps were noted for the illumination of each LED. After the experiments were complete, the raw voltage was exported from Audacity to R, where it was analyzed by WinBeat.R. Threshold was set manually to collect the highest peak, and a count was generated each time the dB threshold level was surpassed. The counts were then averaged out to a per 10 s interval for each insect, and again averaged out for the total number of each species.

**Fig. 9.**
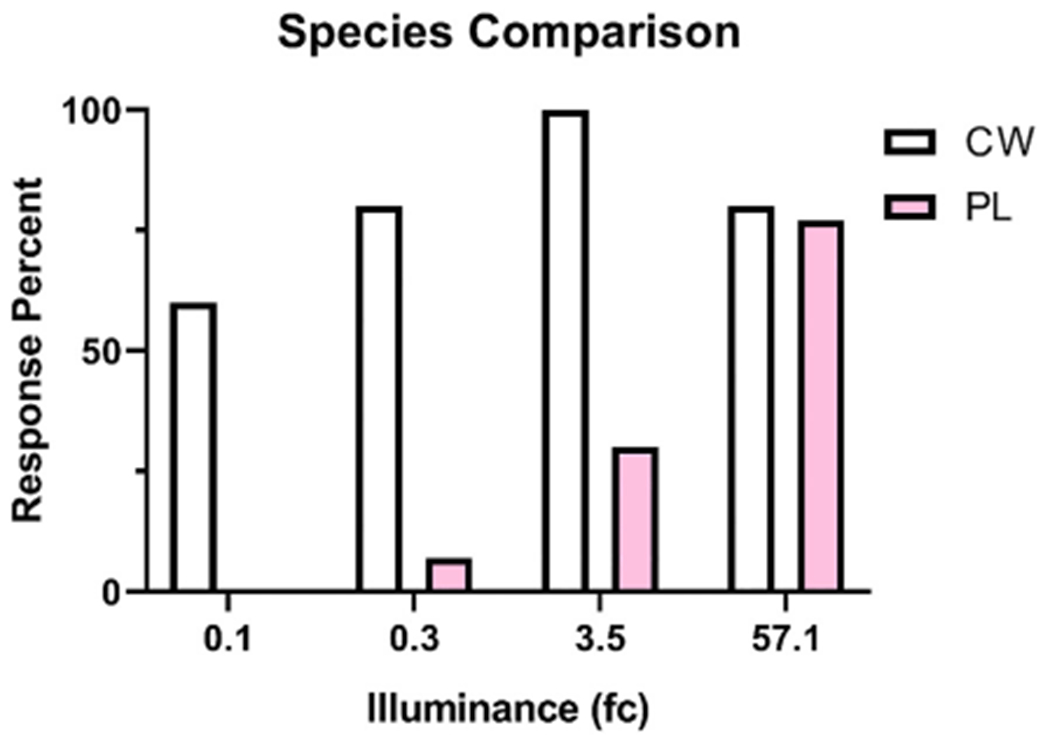
Response rate to illumination following dark adaptation. Response rate was calculated per group as any measureable deflection per intensity (fc). Cabbage whites (CW; white bars) consistently flew to all intensities. Painted ladies (PL; pink bars) did not consistently fly until 57.1 fc. (For interpretation of the references to color in this figure legend, the reader is referred to the web version of this article.)

**Fig. 10.**
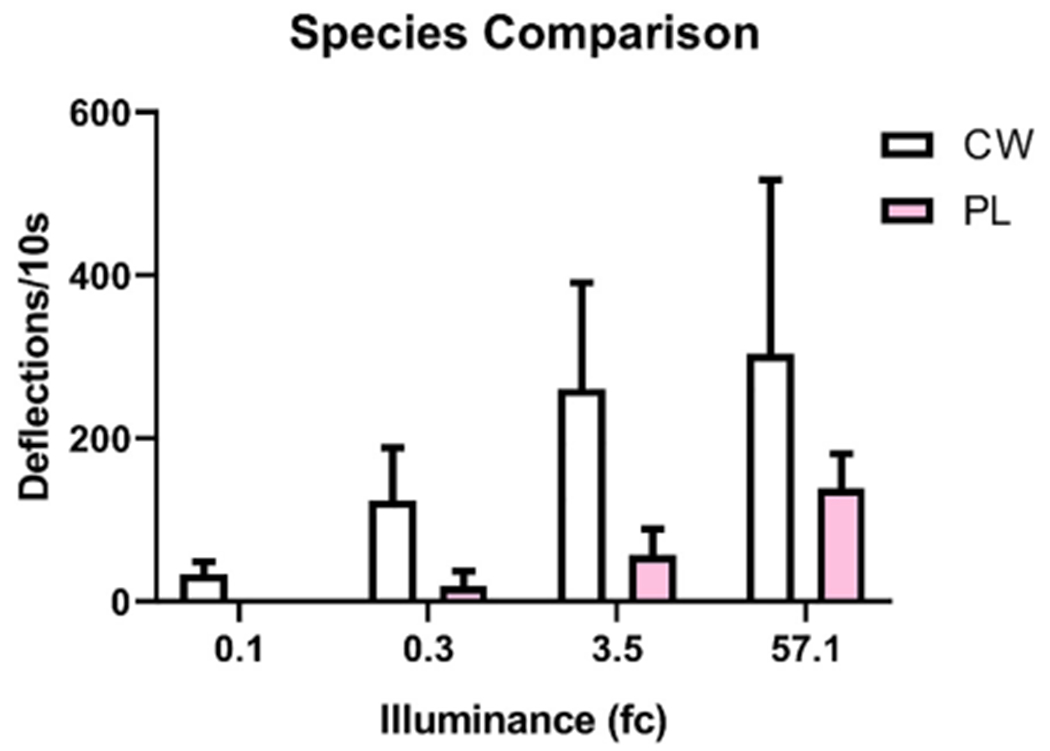
Wingbeat frequency to illumination following dark adaptation. Average movement was calculated as peak deflections of the piezoelectric per 10 s. Cabbage whites (CW; white bars) initiated more wingbeats to illumination than painted ladies (PL; pink bars). (For interpretation of the references to color in this figure legend, the reader is referred to the web version of this article.)

Cabbage whites were more likely to respond to illumination than painted ladies (χ(3) = 79.03, p = 0.0001). A majority of cabbage whites exhibited flying behavior at each light intensity (ranging from 60 to 100%; Fig. 9). While a majority of painted ladies (77%) only flew to the greatest intensity (57.1 fc; Fig. 9). Cabbage whites also generated more deflections than painted ladies (F_(7,64)_ = 3.03, p = 0.008; Fig. 10).

## 8. Conclusions

The purpose of this experiment was to replicate radiation-induced flying experiments conducted by Smith et al. (1963) [8] and use them in the undergraduate teaching laboratory. We created a movement assay using inexpensive, user friendly hardware and software interfaces. Smith et al. (1963) [8] did not have access to microprocessor hardware and associated software and therefore could not investigate wingbeats readily. With either the WingBeat.R script or commercially-available education hardware the wingbeat frequency can be measured with millisecond precision. Modifying the script will allow for further flight analysis (i.e. stroke duration). We were able to expand upon the previous experimental design by quantifying the movement of each insect in response to introduced stimuli.

This apparatus was constructed to measure radiation-induced flying by insects. We hypothesized that insects with a greater sensitivity to light would fly more readily following dark adaptation. Consistent with our hypothesis, we found that cabbage whites were more sensitive than painted ladies to white light across a range of intensities (<0.1–57.1 fc). In fact, painted lady movement did not resemble cabbage white movement until 57.1 fc. Further, cabbage whites showed significant movement to all intensities of light (<0.1–57.1 fc). Painted ladies did not show significant movement until 57.1 fc, the greatest intensity of our experiment. The results of the proof of concept experiment correspond to photosensitivity’s established by previous research [10,12–14]. Thereby, we were able to validate the efficacy of the movement assay.

This apparatus is easy to use, inexpensive, and can be constructed without advanced knowledge of electronics. It can easily be incorporated into existing laboratory infrastructure. Using known electrophysiology hardware and software (iWorx TA modules, Backyardbrains amplifier), we validated the custom R analysis script in the pilot experiment. The experimental design is modular allowing for different stimuli to be introduced to connected insects. As well as modification of hardware components. For example, the Neuron Spikerbox used here could be switched for Heart and Brain Spikerbox (Backyard-brains) or a user-built amplifier [15]. Even the data analysis and graphing can be completed using low-cost alternatives such as the XL Toolbox (www.xltoolbox.net) for Microsoft Excel or with JASP a stand-alone statistics package (http://www.jasp-stats.org/).

The equipment presented here is not limited to the insects or stimuli used in this study; rather, can be modified for stimulus-insect pairing that creates enough movement to cause a deflection in the piezoelectric sensor. Simply changing LEDs on the lightboard could allow for investigation of visual color perception in butterflies [10,12–14] and honeybees [16] or ultraviolet sensitivity in honeybees [16], moths [17], and drosophila [18]. The lightboard could be modified a step further by switching LEDs for piezoelectric buzzers (ex: Adafruit.com product 160) to create a ‘tone board’. This would allow for the testing of ultrasonic sensitivity in many flying insects including antlions and lacewings [19], and noctid moths [20]. Thus, protocols could be developed with this apparatus to investigate a range of insect sensory systems in the context of the undergraduate neuroscience laboratory.

This hardware was designed to provide undergraduate neuroscience students hands-on experience with a classic experiment from the literature [8]. Thus, this hardware can be used to provide context for classroom discussion in several areas ranging from sensory physiology to robotics. The hardware was designed to accompany literature on radiation sensitivity as demonstrate by Smith et al. (1963) [8] but also provides context for the molecular mechanisms for visual transduction [10,12–14] and circuitry [18]. Discussion can easily be extended to insect neuroethology [17,20]. A different approach to discussion could focus on hardware design [1–8] and applications. For example, careful monitoring of complex and multi-segmented movements made by other insects has informed robot design and construction [21,22]. Further discussion could focus on experimental approach. For example, students could compare the trade-offs that need to be considered when selecting a technology. Relevant to our apparatus, a new open-source tool, DeepLabCut, analyses limb and joint movement from videos with high resolution [23]. Students could compare the computing requirements of both systems or the time required to generate data.

## 9. Animal rights

The work here follows the NIH guidelines on animal use.

## Supplementary Material

1
