# Supplementary figures and images for: Movement assay for the undergraduate neuroscience laboratory
Source: HardwareX. 2020 Jan 27;7:e00094. doi: 10.1016/j.ohx.2020.e00094 (PMC7491750; doi:10.1016/j.ohx.2020.e00094)

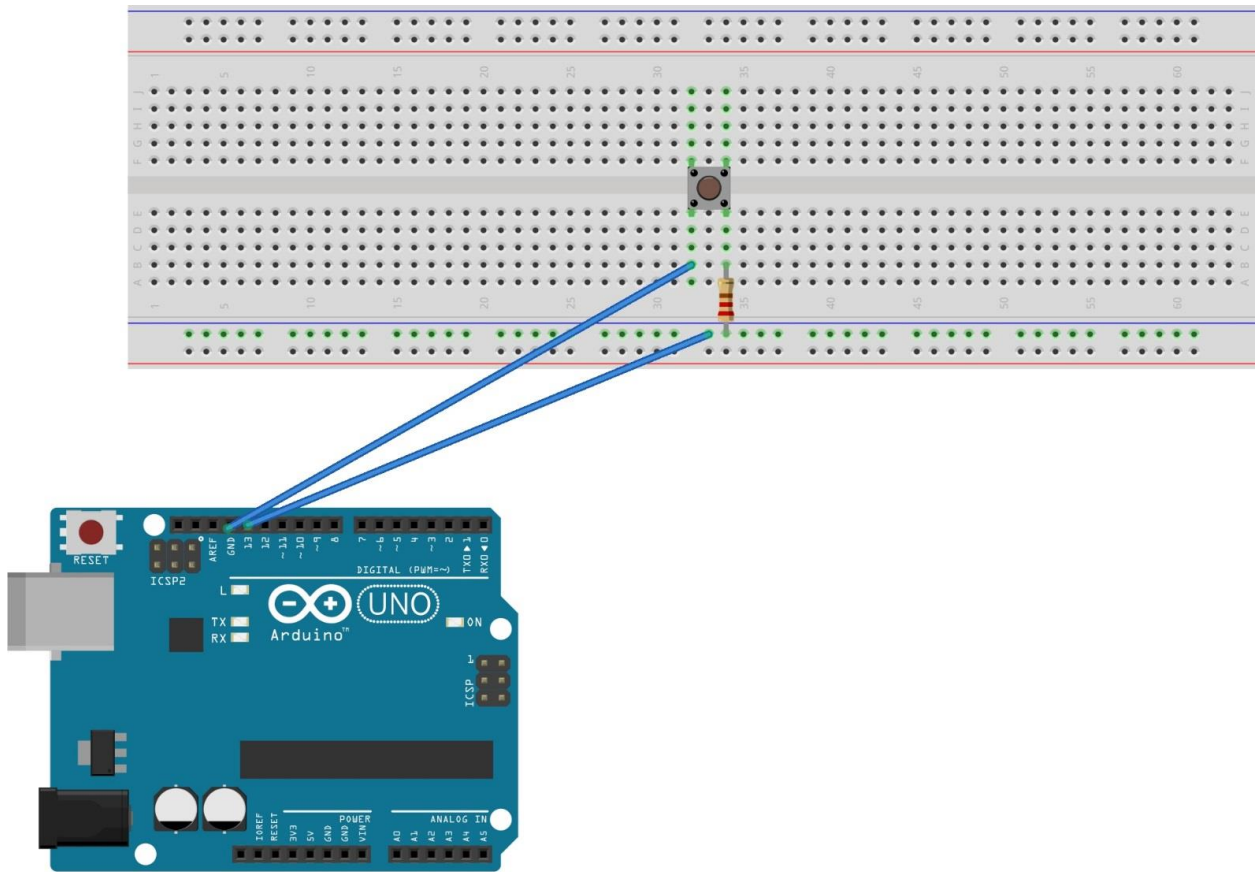

fritzing

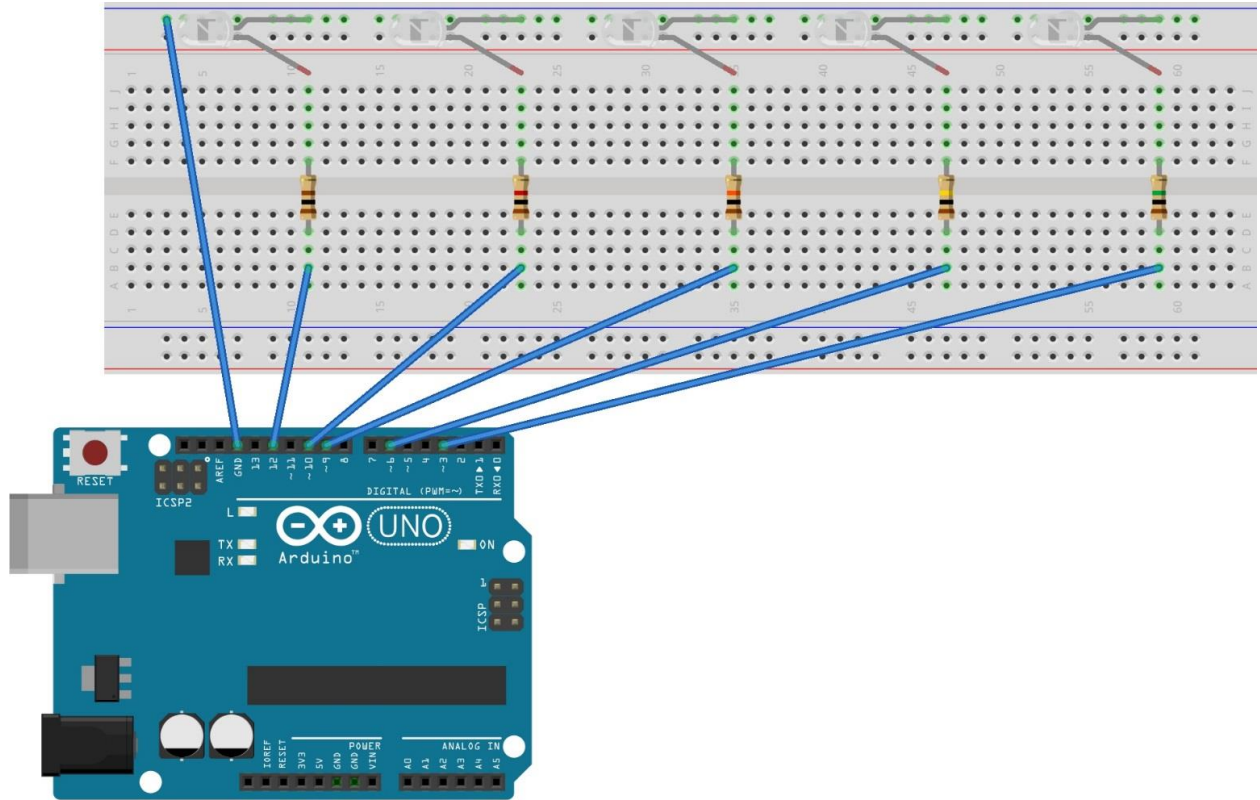

fritzing

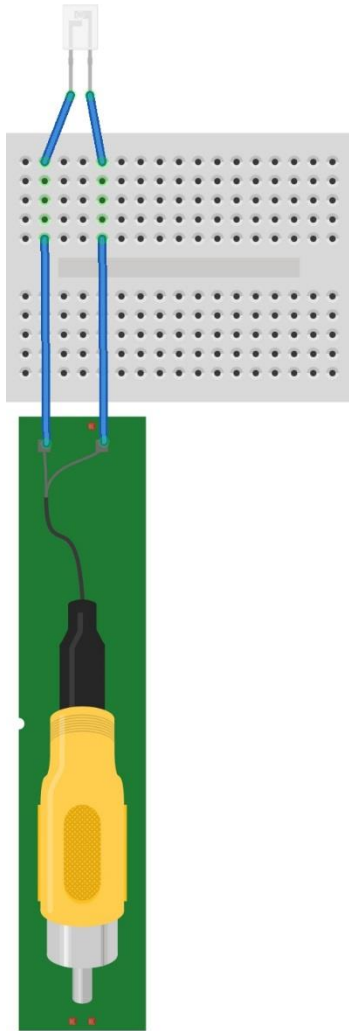

fritzing

Supplement: Supplementary data 1 [file mmc1.pdf]
